# Oral versus intravenous melphalan for autologous stem cell transplantation conditioning in lymphoma and multiple myeloma: a retrospective cohort study

**DOI:** 10.3389/fonc.2026.1923679

**Published:** 2026-10-19

**Authors:** Jingrong Wang, Yimeng Guo, Jin Zhao, Xiaolian Wen, Liping Su, Zhiying Hao, Jing Yang

**Affiliations:** 1 Department of Hematology, Shanxi Province Cancer Hospital/Shanxi Hospital Affiliated to Cancer Hospital, Chinese Academy of Medical Sciences/Cancer Hospital Affiliated to Shanxi Medical University, Taiyuan, China; 2 Department of Pharmacy, Shanxi Province Cancer Hospital/Shanxi Hospital Affiliated to Cancer Hospital, Chinese Academy of Medical Sciences/Cancer Hospital Affiliated to Shanxi Medical University, Taiyuan, China

**Keywords:** autologous stem cell transplantation, intravenous, lymphoma, melphalan, multiple myeloma, oral

## Abstract

**Objective:**

To compare oral (PO) and intravenous (IV) melphalan as conditioning regimens for autologous stem cell transplantation in patients with lymphoma or multiple myeloma (MM).

**Methods:**

A retrospective propensity score-matched cohort analysis of 106 patients (76 with lymphoma and 30 with MM) was conducted to compare engraftment outcomes, adverse events, and survival between PO and IV melphalan conditioning. The primary endpoint was 3-year progression-free survival.

**Results:**

The BEAM (carmustine, etoposide, cytarabine, and melphalan) regimen is the standard conditioning regimen for lymphoma and a single-agent melphalan was administered for MM. In patients with lymphoma, IV melphalan was associated with faster neutrophil engraftment than PO melphalan (10 vs 12 days, *p* = 0.025). In MM, IV melphalan was associated with faster platelet (PLT) engraftment (11 vs 14 days, *p* = 0.021). The PO group had higher grade 3–4 anemia (37.74% vs 18.87%, *p* = 0.031) but lower febrile neutropenia (35.85% vs 56.60%, *p* = 0.032) and mucositis (7.55% vs 33.96%, *p* = 0.001). In the PO group, grade 3–4 anemia correlated with delayed PLT engraftment (62.5% vs 21.4%, *p* = 0.008). Compared with IV melphalan, divided-dose oral melphalan was associated with lower rates of diarrhea (18.87% vs 37.74%) and oral mucositis (7.55% vs 33.96%), with an overall gastrointestinal toxicity rate (defined as at least one gastrointestinal adverse event) of 21.7% in the PO group versus 56.60% in the IV group. Complete response rates and 3-year survival outcomes were comparable between the groups.

**Conclusion:**

Oral melphalan is a pragmatic alternative when IV melphalan is unavailable, with comparable efficacy and a distinct toxicity profile. Given variable bioavailability, it should not be considered bioequivalent to IV melphalan. Prospective pharmacokinetic studies are warranted to validate these findings.

## 1 Introduction

Although non-Hodgkin lymphoma (NHL) and Hodgkin lymphoma (HL) are generally sensitive to chemotherapy, many patients relapse or have refractory disease following first-line treatment. High-dose chemotherapy followed by autologous stem cell transplantation (ASCT) improves disease-free and overall survival (OS) compared with conventional salvage therapy (1–5).

The BEAM (carmustine, etoposide, cytarabine, and melphalan) regimen is the standard conditioning regimen for lymphoma ASCT (3–5). However, shortages and the high cost of intravenous (IV) melphalan have prompted many Chinese institutions to substitute oral melphalan tablets (2 mg/tablet) (6–8).

Multiple myeloma (MM) treatment has advanced with novel agents (9), but high-dose chemotherapy followed by ASCT remains standard care for eligible patients younger than 65 years (10–13). Oral melphalan has similarly been adopted due to IV shortages and cost constraints. Notably, oral melphalan bioavailability is highly variable (20–60%) owing to erratic absorption and first-pass metabolism (14, 15).

Despite growing use, systematic comparisons of IV melphalan, the standard formulation, and oral (PO) melphalan, the alternative formulation, for ASCT conditioning remain limited. This retrospective study compared hematopoietic reconstitution, treatment-related adverse events, and survival between formulations in Chinese patients with lymphoma and MM undergoing ASCT.

## 2 Materials and methods

### 2.1 Patients

A single-center, hypothesis-generating retrospective analysis included patients with lymphoma or MM prior to ASCT at the Department of Hematology, Shanxi Province Cancer Hospital, between January 2015 and December 2023. Of 164 patients who were initially screened, 112 had lymphoma (NHL, *n* = 82; HL, *n* = 30) and 52 had MM. ALL patients were eligible for PSM. Using 1:1 nearest-neighbor matching with a caliper width of 0.2, 106 patients were successfully matched and included in the final analysis, comprising 76 patients with lymphoma (NHL, n = 56; HL, *n* = 20) and 30 patients with MM (*n* = 15 per group). To increase statistical power for subsequent analyses, patients with NHL and HL were combined into a single lymphoma cohort (IV group, *n* = 38; PO group, *n* = 38).

There were 82 cases of non-Hodgkin’s lymphoma. 26 cases did not match. Among them, 15 cases were DLBCL, 2 cases each were T lymphoblastic lymphoma, mantle cell lymphoma, and large B-cell lymphoma. The remaining cases were follicular lymphoma, small cell lymphoma, liver and spleen T-cell lymphoma, peripheral T, NK/T, each with 1 case. In total, there were 26 cases. 10 cases of Hodgkin’s lymphoma did not match. They were 5 cases of nodular sclerosis type, 3 cases of mixed cell type, 1 case of lymphocyte depletion type, and 1 case without classification. In total, there were 10 cases. 22 cases of MM did not match. They were 8 cases of IgA, 2 cases of IgD, 7 cases of light chain κ, and 4 cases of light chain λ.

Approval for the study was obtained from the institutional ethics committee (approval number: KY2025022). Follow-up for survival outcomes was conducted until September 2024.

#### 2.1.1 Inclusion criteria

Inclusion criteria for the lymphoma group were: (1) newly diagnosed high-risk non- HL (defined as an International Prognostic Index (IPI) score ≥ 3, the presence of high-risk cytogenetic features such as MYC/BCL2 double expression or *TP53* variant, or primary refractory NHL or relapsed/refractory NHL and HL achieving either complete remission (CR) or partial remission (PR); (2) age between 18 and 70 years; (3) Eastern Cooperative Oncology Group performance status of 0–2; (4) adequate cardiac, hepatic, pulmonary, and renal function before transplantation (cardiac function: left ventricular ejection fraction ≥ 50%; hepatic function: ALT/AST ≤ 2 × the upper limit of normal (ULN) and total bilirubin ≤ 1.5 × ULN; pulmonary function: forced vital capacity ≥ 60% of the predicted value and blood oxygen saturation ≥ 95%; renal function: estimated glomerular filtration rate, calculated using the CKD-EPI formula, ≥ 40 mL/min/1.73 m²); and (5) BEAM conditioning.

Inclusion criteria for the MM group were: (1) fulfillment of the diagnostic criteria for MM; (2) age ≤ 70 years; (3) absence of severe organ dysfunction, with endogenous creatinine clearance (calculated using the CKD-EPI formula) ≥ 40 mL/min/1.73 m²; (4) achievement of at least PR following induction therapy; and (5) high-dose melphalan conditioning.

Exclusion criteria were: (1) a history of treatment for a prior malignancy; (2) abnormal organ function; (3) mental disorders; (4) dose adjustment within the conditioning regimen; (5) incomplete or missing key clinical data; or (6) loss to follow-up within 100 days after ASCT.

#### 2.1.2 Conditioning regimens

Conditioning differed by disease type and melphalan formulation. For patients with lymphoma (BEAM regimen), carmustine 300 mg/m² was administered intravenously on day −6, etoposide 200 mg/m² intravenously on days −5 to −2, and cytarabine 200 mg/m² intravenously every 12 hours on days −5 to −2. Melphalan was administered on day −1 at a total dose of 140 mg/m². In the IV group, the entire dose was administered as a single IV infusion. In the PO group, the total dose was divided into two equal portions and administered approximately 12 hours apart (e.g., 70 mg/m² in the morning and 70 mg/m² in the evening) on day −1 under fasting conditions (at least 2 hours before or after meals).

For patients with MM, high-dose melphalan was administered at a total dose of 200 mg/m² over two consecutive days (days −3 and −2), followed by a rest day on day −1. In the IV group, the daily dose (100 mg/m²) was administered as a single IV infusion on each day. In the PO group, each daily dose was divided into two equal portions administered approximately 12 hours apart on each day (e.g., 50 mg/m² twice daily on days −3 and −2), resulting in a total of four oral doses over the 2-day period. All oral doses were administered under fasting conditions to minimize variability in absorption. For all patients, autologous peripheral blood stem cells were reinfused on day 0, approximately 24 hours after the final melphalan dose in patients with lymphoma (day −1) and approximately 48 hours after the final melphalan dose in patients with MM (day −2). Red blood cell transfusion was indicated for a hemoglobin level < 70 g/L or the presence of clinically significant symptoms of anemia. Platelet (PLT) transfusion was indicated for a PLT count < 20 × 10^9^/L or evidence of a bleeding tendency. The transfusion strategy was standardized across both groups.

Assignment to IV or PO melphalan was based on patient preference following discussion of the off-label use of oral melphalan, financial considerations, and IV melphalan availability, which was subject to intermittent shortages in China during the study period.

### 2.2 Definitions and outcome assessment

Neutrophil engraftment was defined as the first of three consecutive days on which the absolute neutrophil count (ANC) was ≥ 0.5 × 10^9^/L, without subsequent decline and without granulocyte colony-stimulating factor (G-CSF) support. G-CSF absence was defined as no G-CSF administration during hematopoietic reconstitution, or only prophylactic G-CSF administration within 72 hours after transplantation, provided the engraftment criterion was met after drug withdrawal. PLT engraftment was defined as the first of seven consecutive days with PLT count was > 20 × 10^9^/L without transfusion.

Lymphoma response was assessed per the 2007 Revised Response Criteria for Malignant Lymphoma. MM response was assessed according to the International Myeloma Working Group criteria, which classify responses as stringent complete response, CR, very good partial response (VGPR), partial response, stable disease, and progressive disease. OS was the time from ASCT to death from any cause. Patients who were alive at the last follow-up were censored. Progression-free survival (PFS) was defined as the time from ASCT to the first occurrence of disease progression, relapse, or death from any cause. Patients who were alive and progression-free at the last follow-up were censored. Febrile neutropenia was defined as an ANC < 0.5 × 10^9^/L accompanied by an axillary temperature ≥ 38.0 °C or a temperature ≥ 37.7 °C persisting for more than 1 hour. Adverse events were assessed according to the Common Terminology Criteria for Adverse Events, version 5.0.

### 2.3 Supportive care

The antiemetic regimen is the same as the intravenous group. The Beam regimen: Palonosetron 0.25mg iv, d-6 + Forsapitant 150mg iv, d-6 + Dexamethasone 8mg iv once daily, d-6, d-5, d-4, d-3; The mm regimen: Palonosetron 0.25mg iv, + Forsapitant 150mg iv, pre-treatment d-3 + Dexamethasone 8mg iv once daily, d-3, d-2, d-1, once daily. All antiemetic drugs were administered 30 minutes before the start of chemotherapy on the same day; for cases of explosive vomiting, metoclopramide could be used temporarily for symptomatic treatment.

Prevention of pneumocystis pneumonia: From the start of pre-treatment, take oral compound sulfamethoxazole (TMP-SMX), with TMP at 80 mg and SMX at 400 mg, 2 tablets, twice a day until 3 months after transplantation. After stem cell infusion, the ANC is 1.0 - 1.5 × 10^9^/L, and the G-SCF dose is 5 μg/kg/d, subcutaneous injection, once a day. Indications for discontinuation:

For 3 consecutive days, ANC ≥ 0.5 × 10^9^/L. Prevention of gastrointestinal source infection: Gentamicin 80 mg, oral, 3 times a day; Norfloxacin capsules 0.2 g each time, oral, 3 times a day; The starting time of antifungal treatment is on the day of pre-treatment, administration: Fluconazole dose: 200–400 mg qd, intravenously. Discontinuation: When neutrophils recover (ANC > 0.5 × 10^9^/L).

### 2.4 Statistical analysis

All statistical tests were two-tailed, and a significance level of *p* < 0.05 was used to determine statistical significance. To reduce selection bias, propensity score matching (PSM) was performed at a 1:1 ratio using nearest-neighbor matching with a caliper width of 0.2 standard deviations of the logit of the propensity score. Covariates included age, sex, disease stage, pre-transplant disease status, and time from diagnosis to transplantation. Balance between the groups after matching was assessed using standardized mean differences (SMDs), with an SMD < 0.1 considered indicative of good balance. For variables with residual imbalance (SMD ≥ 0.2) after PSM, a dual approach to confounding adjustment was applied through stratified analyses and multivariable correction. First, subgroup analyses were conducted according to the imbalanced variables. Subsequently, all imbalanced variables were included in the Cox regression model for multivariable adjustment to ensure the robustness of the results.

Given the limited sample size, 1:1 PSM can further reduce power and increase overmatching risk. To reduce these risks, only clinically relevant covariates were included in the matching model. Post-matching balance was assessed using SMDs, with an SMD ≥ 0.2 considered indicative of residual imbalance. All variables with an SMD ≥ 0.2, together with established prognostic factors, were subsequently adjusted for multivariable analyses to minimize confounding. However, given the exploratory nature of the study and the reduced sample size after matching, the results, particularly those of the subgroup analyses, should be interpreted with caution.

Statistical analyses were conducted using SPSS software (version 29.0). Patient demographic and disease characteristics were summarized using medians, ranges, frequencies, and percentages. Normality of continuous variables was assessed using the Shapiro–Wilk test. All continuous variables were found to be non-normally distributed (Shapiro–Wilk test, *p* < 0.05) and were therefore compared using the Mann–Whitney U test. Categorical variables were compared using the chi-square test or Fisher’s exact test, as appropriate. The statistical test used for each analysis was clearly indicated in the corresponding tables.

PFS was the time from ASCT to disease progression, relapse, or death. Patients who were alive and progression-free at the last follow-up were censored. OS was the time from ASCT to death from any cause. Survival curves were generated by the Kaplan–Meier method and compared by the log-rank test. Multivariable Cox proportional hazards models assessed the independent effect of the route of administration on PFS and OS after adjustment for potential confounders, including all variables with an SMD ≥ 0.2 after matching and clinically established prognostic factors (e.g., pre-ASCT disease status and cytogenetic risk). HRs and 95% CIs were calculated. Given the limited number of events in certain subgroups (e.g., HL, *n* = 20), the corresponding survival analyses should be considered exploratory.

## 3 Results

### 3.1 Patient characteristics

Of 161 patients who underwent ASCT between January 2015 and December 2023 and were initially screened, 112 had lymphoma (NHL, *n* = 82; HL, *n* = 30) and 49 had MM. After excluding dose adjustments, incomplete data, or loss to follow-up, 143 were eligible for PSM. Using 1:1 nearest-neighbor matching with a caliper width of 0.2, 106 patients were successfully matched and included in the final analysis, comprising 76 patients with lymphoma (NHL, n = 56; HL, *n* = 20) and 30 patients with MM (*n* = 15 per group). To increase statistical power for subsequent analyses, patients with NHL and HL were combined into a single lymphoma cohort (IV group, *n* = 38; PO group, *n* = 38).

Post-matching balance diagnostics showed that most baseline characteristics were comparable but some imbalances persisted after PSM (**Tables 1**–**3**). In the NHL cohort, a statistically significant difference in sex distribution was observed between the IV and PO groups (82.1% vs 57.1% male, *p* = 0.042), with a corresponding large standardized mean difference (SMD = 0.54). Residual imbalances were observed for disease status (SMD = 0.24) in patients with NHL; disease status (SMD = 0.30) and time to transplantation (SMD = −0.38) in patients with HL; and Durie–Salmon stage (SMD = 0.25) and M-component type (SMD = −0.58) in patients with MM, although these differences did not reach statistical significance (*p* > 0.05). To reduce the potential confounding effects of these imbalances, all variables with an SMD ≥ 0.2, together with clinically established prognostic factors, were subsequently adjusted for in multivariable analyses.

**Table 1:** Baseline characteristics of nhl patients after propensity score matching.

Characteristic	IV (*n* = 28)	PO (*n* = 28)	*p*–value	SMD
Median age, years (range)	43 (19–64)	35 (18–65)	0.818	**0.53**
Sex, *n* (%)			0.042	0.54
Male	23 (82.1)	16 (57.1)		
Female	5 (17.9)	12 (42.9)		
Disease type, *n* (%)			0.408	
B cell lymphoma	16 (57.1)	19 (67.9)		**-0.22**
T- and NK-cell lymphoma	12 (42.9)	9 (32.1)		**0.22**
Ann Arbor stage, *n* (%)			1.000	
Stage I-II	6 (21.4)	5(17.9)		0.09
Stage III- IV	22 (78.6)	23 (82.1)		-0.09
IPI scores, *n* (%)			1.000	
1-3	20 (71.4)	20 (71.4)		0.00
4-5	8 (28.6)	8 (28.6)		0.00
Disease status before ASCT, *n* (%)			0.670	0.24
Complete remission (CR)	26 (92.9)	24 (85.7)		
Partial remission (PR)	2 (7.1)	4 (14.3)		
Newly diagnosed or relapsed/refractory disease			0.592	0.14
newly diagnosed	16 (57.1)	14 (50.0)		
relapsed/refractory disease	12 (42.9)	14 (50.0)		
Time from diagnosis to transplant, median (range), months	8 (5-60)	9 (5-84)	0.166	-0.08

Data are presented as median (range) or number of patients (%). *P*-values were derived from the Mann-Whitney U test (continuous variables), Chi-square or Fisher’s exact test (categorical variables). Standardized mean differences (SMD) were used to assess post-matching balance, with SMD <0.1 indicating good balance, 0.1-0.2 indicating acceptable small differences, and ≥ 0.2 indicating residual imbalance. Bold SMD values indicate variables with residual imbalance (SMD ≥ 0.2). The SMD value of 0.24 for “Disease status before ASCT” represents the standardized mean difference calculated for the overall status variable (CR vs PR as a binary category). IPI, International Prognostic Index.

**Table 2:** Baseline characteristics of HL patients after propensity score matching.

Characteristic	IV (*n* = 10)	PO (*n* = 10)	*p*–value	SMD
Median age, years (range)	26 (17-38)	25 (17-36)	0.171	0.09
Sex, *n* (%)			1.000	0.000
Male	6 (60.0)	6 (60.0)		
Female	4 (40.0)	4 (40.0)		
Ann Arbor stage, *n* (%)			1.000	0.000
I–II	2 (20.0)	2 (20.0)		
III–IV	8 (80.0)	8 (80.0)		
Disease status before ASCT, *n* (%)			1.000	0.30
Complete remission (CR)	9 (90.0)	8 (80.0)		
Partial remission (PR)	1 (10.0)	2 (20.0)		
Time from diagnosis to transplant, median (range), months	11 (7–192)	22 (12–66)	0.341	**-0.38**

Data are presented as median (range) or number of patients (%). *P*–values were derived from the Mann-Whitney U test (continuous variables) or Fisher’s exact test (categorical variables). Standardized mean differences (SMD) were used to assess post-matching balance, with SMD <0.1 indicating good balance, 0.1-0.2 indicating acceptable small differences, and ≥ 0.2 indicating residual imbalance. Bold SMD values indicate variables with residual imbalance (SMD ≥ 0.2).

**Table 3:** Baseline characteristics of MM patients after propensity score matching.

Characteristic	IV (*n* = 15)	PO (*n* = 15)	*p*-value	SMD
Median age, years (range)	56 (35-67)	54 (38-61)	0.898	0.13
Sex, *n* (%)			1.000	0.14
Male	10 (66.7)	9 (60.0)		
Female	5 (33.3)	6 (40.0)		
Durie-Salmon stage, *n* (%)			0.432	
I	2 (13.3)	0 (00.0)		**0.25**
II	1 (6.7)	2 (13.3)		-0.12
III	12 (80.0)	13 (86.7)		-0.20
M component			0.170	
IgG	8 (53.3)	12 (80.0)		**-0.58**
Other types	7 (46.7)	3 (20.0)		**0.58**
Disease status before ASCT, *n* (%)			0.340	
Complete remission (CR)	5 (33.3)	6 (40.0)		-0.14
Very good partial response (VGPR)	8 (53.4)	7 (46.7)		0.13
Partial remission (PR)	2 (13.3)	2 (13.3)		0.00
Cytogenetics/FISH at diagnosis, n (%)			1.000	0.000
High risk	7 (46.7)	7 (46.7)		
Low risk	8 (53.3)	8 (53.3)		
Time from diagnosis to transplant, median (range), months	9 (6-12)	9 (5-19)	0.139	0.000

Data are presented as median (range) or number of patients (%). *P*-values were derived from the Mann-Whitney U test (continuous variables) or Fisher’s exact test (categorical variables). Standardized mean differences (SMD) were used to assess post-matching balance, with SMD < 0.1 indicating good balance, 0.1-0.2 indicating acceptable small differences, and ≥ 0.2 indicating residual imbalance. Bold SMD values indicate variables with residual imbalance (SMD ≥ 0.2). VGPR, very good partial response.

### 3.2 Hematopoietic reconstitution

All patients achieved hematopoietic reconstitution. In the lymphoma cohort, the median number of reinfused CD34+ cells was 6.10 × 10^6^/kg in the IV group and 5.60 × 10^6^/kg in the PO group (*p* = 0.499). The corresponding values in the MM cohort were 4.63 × 10^6^/kg and 3.24 × 10^6^/kg, respectively (*p* = 0.461). Red blood cell and PLT transfusion requirements were comparable between the groups (**Tables 4**, **5**).

**Table 4:** Hematopoietic reconstitution in lymphoma patients.

Parameter	IV group (*n* = 38)	PO group (*n* = 38)	*p*-value
Median number of reinfused CD34 + cells, ×10^6^/kg (range)	6.10 (2.02-29.12)	5.60 (1.00-27.27)	0.499
Median number of RBC infusions, units (range)	0.00 (0.00-8.00)	0.00 (0.00-12.00)	0.298
Median number of PLT infusions, units (range)	3.00 (1.00-15.00)	2.00 (0.00-15.00)	0.232
Median time to ANC > 0.5×10^9^/L, days (IQR)	10.00 (9.00-11.00)	12.00 (11.00-13.00)	**0.025**
Median time to PLTc > 20×10^9^/L, days (IQR)	11.00 (10.00-12.00)	13.00 (12.00-14.00)	0.224

Data are presented as median (interquartile range, IQR) unless otherwise specified. *P-*values were derived from the Mann-Whitney U test. Bold p-values indicate statistical significance (*p* < 0.05). Neutrophil engraftment was defined as ANC ≥0.5×10^9^/L for three consecutive days; platelet engraftment was defined as PLT >20×10^9^/L for seven consecutive days without transfusion support.

**Table 5:** Hematopoietic reconstitution in MM patients.

Parameter	IV (*n* = 15)	PO (*n* = 15)	*p*-value
Median number of reinfused CD34 + cells, ×10^6^/kg (range)	4.63 (2.20–8.11)	3.24 (1.33–19.24)	0.461
Median number of RBC infusions, units (range)	0.00 (0.00–4.00)	0.00 (0.00-0.00)	0.775
Median number of PLT infusions, units (range)	2.00 (1.00–6.00)	1.00 (0.00–4.00)	0.061
Median time to ANC > 0.5×10^9^/L, days (IQR)	11.00 (10.00–12.00)	11.00 (10.00–12.00)	0.325
Median time to PLTc > 20×10^9^/L, days (IQR)	11.00 (10.00–12.00)	14.00 (13.00–15.00)	**0.021**

Data are presented as median (interquartile range, IQR) unless otherwise specified. *P*–values were derived from the Mann–Whitney U test. Bold *p*–values indicate statistical significance (*p* < 0.05). Neutrophil engraftment was defined as ANC ≥ 0.5×10^9^/L for three consecutive days; platelet engraftment was defined as PLT > 20×10^9^/L for seven consecutive days without transfusion support.

Regarding engraftment kinetics, more rapid engraftment was observed in the IV group than in the PO group. The median time for neutrophil engraftment in the myeloma group was 11 (IQR: 8.00-16.00) days, in the non-Hodgkin’s lymphoma group it was 10 (IQR: 8.00-16.00) days, and in the Hodgkin’s lymphoma group it was 10.5 [IQR: 8.00-17.00] days. The median time for platelet engraftment was 12 (IQR: 8.00-18.00) days in the myeloma group, 12 (IQR: 8.00-37.00) days in the non-Hodgkin’s lymphoma group, and 13 (IQR: 7.00-28.00) days in the Hodgkin’s lymphoma group. Among patients with lymphoma, the median time to neutrophil engraftment was significantly shorter in the IV group than in the PO group (10.00 [IQR: 9.00–11.00] days vs 12.00 [IQR: 11.00–13.00] days; *p* = 0.025). In patients with MM, the median time to neutrophil engraftment was similar between the groups (11.00 [IQR: 10.00–12.00] days in both groups; *p* = 0.325). PLT engraftment occurred later than neutrophil engraftment in both cohorts. A tendency of delay in PLT engraftment was observed in the PO group, with median times of 11.00 (IQR: 10.00–12.00) days versus 13.00 (IQR: 12.00–14.00) days (*p* = 0.224) in patients with lymphoma, and 11.00 (IQR: 10.00–12.00) days versus 14.00 (IQR: 13.00–15.00) days (*p* = 0.021) in patients with MM, for the IV and PO groups, respectively (**Tables 4**, **5**).

### 3.3 Treatment–related adverse events

Adverse events are summarized in **Table 6**. Grade 3–4 anemia was more frequent in PO than IV (37.74% vs 18.87%; *p* = 0.031), whereas febrile neutropenia was less frequent in PO (35.85% vs 56.60%; *p* = 0.032). In the PO group, grade 3–4 anemia was observed significantly more frequently among patients with delayed PLT engraftment than normal PLT engraftment (62.5% vs 21.4%; *p* = 0.008).

**Table 6:** Treatment–related adverse events in all patients after propensity score matching.

Toxicities	IV group (*n* = 53)	PO group (*n* = 53)	*p*–value
Hematologic toxicity			
Grade 3–4 Anemia, n (%)	10 (18.87)	20 (37.74)	**0.031**
Febrile neutropenia, n (%)	30 (56.60)	19 (35.85)	**0.032**
Non–hematologic toxicity			
Nausea/vomiting, (Any Grade), *n* (%)	46 (86.79)	41 (77.36)	0.205
Diarrhea, (Any Grade), n (%)	20 (37.74)	10 (18.87)	**0.031**
Oral mucositis, (Any Grade), n (%)	18 (33.96)	4 (7.55)	**0.001**
Elevated transaminase, (Any Grade), n (%)	3 (5.66)	7 (13.21)	0.184
Electrolyte disorders	4 (7.55)	2 (3.77)	0.674
Death prior to day 100, *n* (%)	0	0	–

Data are presented as number of patients (%). *P*–values were derived from the Chi–square test or Fisher’s exact test. Bold *p*–values indicate statistical significance (*p* < 0.05). Toxicities were graded according to CTCAE version 5.0.

Regarding nonhematologic toxicities, gastrointestinal adverse events were more common in the IV group. Significantly higher incidences of diarrhea (37.74% vs 18.87%; *p* = 0.031) and oral mucositis (33.96% vs 7.55%; *p* = 0.001) were observed in the IV group than in the PO group. The overall proportion of patients who experienced at least one gastrointestinal adverse event (diarrhea or oral mucositis) was 56.60% (30/53) in the IV group compared with 22.6% (12/53) in the PO group. Among patients who developed oral mucositis, most were in CR at the time of transplantation (83.33% in the IV group and 100% in the PO group; **Supplementary Table S1**).

Other nonhematologic adverse events, including nausea/vomiting, elevated transaminase levels, and electrolyte disturbances, were comparable between groups and were predominantly grade 1–2 in severity. No cardiac, renal, or central nervous system toxicities were observed. The 100-day transplantation-related mortality rate was 0%.

### 3.4 Survival outcomes

Response evaluation 100 days after ASCT demonstrated no statistically significant differences in CR rates between the IV and PO groups in either the lymphoma cohort (92.11% vs 81.58%; *p* = 0.123) or the MM cohort (40.00% vs 40.00%; *p* = 1.000) (**Tables 7**, **8**). The numbers and proportions of censored cases were added to **Tables 7**, **8**, and the reason for censoring was specified as “no disease progression/death at the last follow-up.”

**Table 7:** Main survival outcomes in lymphoma patients.

Parameter	IV group (*n* = 38)	PO group (*n* = 38)	*p*–value
Median follow–up (months)	42(5–72)	73(4–128)	<0.001
Median PFS (months)	Not reached	Not reached	0.592
3–year PFS rate (KM method)	78.9% (30/38)	81.6% (31/38)	0.773
Median OS (months)	Not reached	Not reached	0.824
3–year OS rate (KM method)	89.5% (34/38)	86.8% (33/38)	0.722

Data are presented as median (range) or estimated rate (number of events/total). *P*–values for survival times were derived from the Log–rank (Mantel–Cox) test; *p*–values for survival rates were derived from the Chi–square test. At day 100 post–ASCT, the complete remission rates were 92.11% vs 81.58% (*p* = 0.123) for lymphoma patients in the IV and PO groups, respectively. PFS, progression–free survival; OS, overall survival.

**Table 8:** Main survival outcomes in MM patients.

Parameter	IV group (*n* = 15)	PO group (*n* = 15)	*p*–value
Median follow–up (months)	43 (21–69)	46 (19–95)	0.198
Median PFS (months)	43.0 (28.78–57.22)	Not reached	0.187
3–year PFS rate (KM method)	66.7% (10/15)	73.3% (11/15)	1.000
Median OS (months)	Not reached	Not reached	0.961
3–year OS rate (KM method)	86.7% (13/15)	80.0% (12/15)	0.672

Data are presented as median (range) or estimated rate (number of events/total). *P*–values for survival times were derived from the Log–rank (Mantel–Cox) test; *p*–values for survival rates were derived from the Chi–square test. At day 100 post–ASCT, the complete remission rates were 40.00% vs 40.00% (*p* = 1.000) for MM patients in the IV and PO groups, respectively. PFS, progression–free survival; OS, overall survival.

Medium- to long-term survival outcomes were comparable between the IV and PO groups. In the lymphoma cohort, despite a significantly longer median follow-up in the PO group than in the IV group (73 [IQR: 45.00–98.00] months vs 42 [IQR: 28.00–56.00] months; *p* < 0.001), neither median PFS nor median OS was reached in either group. No significant differences were observed in the estimated 3-year PFS rates (78.9% vs 81.6%; *p* = 0.773) or 3-year OS rates (89.5% vs 86.8%; *p* = 0.722) (**Tables 7**, **8**).

In the high-risk lymphoma subgroup (IPI ≥ 3), the estimated 3-year PFS rates were 70.0% and 73.3% (*p* = 0.892), and the estimated 3-year OS rates were 80.0% and 83.3% (*p* = 0.915), in the IV and PO groups, respectively, with no significant differences observed between the groups. In the MM cohort, median follow-up durations were comparable between the IV and PO groups (43 [IQR: 32.00–54.00] months vs 46 [IQR: 35.00–58.00] months; *p* = 0.198). No statistically significant differences were observed in median PFS (43.0 [IQR: 35.00–51.00] months vs not reached; *p* = 0.187), median OS (not reached in either group; *p* = 0.961), estimated 3-year PFS rates (66.7% vs 73.3%; *p* = 1.000), or estimated 3-year OS rates (86.7% vs 80.0%; *p* = 0.672) (**Table 8**).

A PO subgroup analysis comparing patients with and without diarrhea showed no significant association between diarrhea and survival outcomes in either patients with lymphoma (**Supplementary Table S2**) or those with MM (**Supplementary Table S3**), suggesting that gastrointestinal toxicity may not be a surrogate efficacy marker.

The log-rank test χ² values, *p* values, and 95% CIs were added to **Figures 1**, **2**. Censored observations were marked on the survival curves to facilitate visualization of the distribution of censored data. Figure legends indicate that curves were generated by Kaplan–Meier, between-group comparisons used the log-rank test with the IV group as reference, and adjustments were made for age, disease status, and other potential confounding factors. Survival curves for patients with lymphoma are presented in **Figure 1**, and those for patients with MM are presented in **Figure 2**.

**Figure 1:**
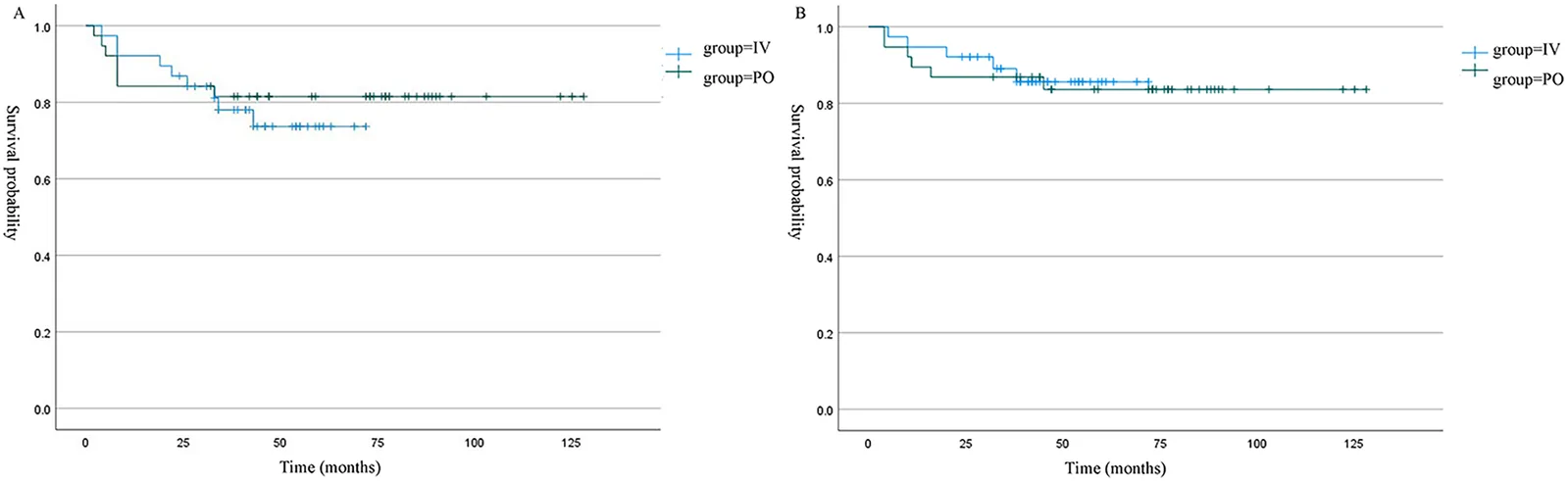
Kaplan-Meier estimates of progression-free survival **(A)** and overall survival **(B)** in lymphoma patients according to melphalan formulation (IV vs. PO).

**Figure 2:**
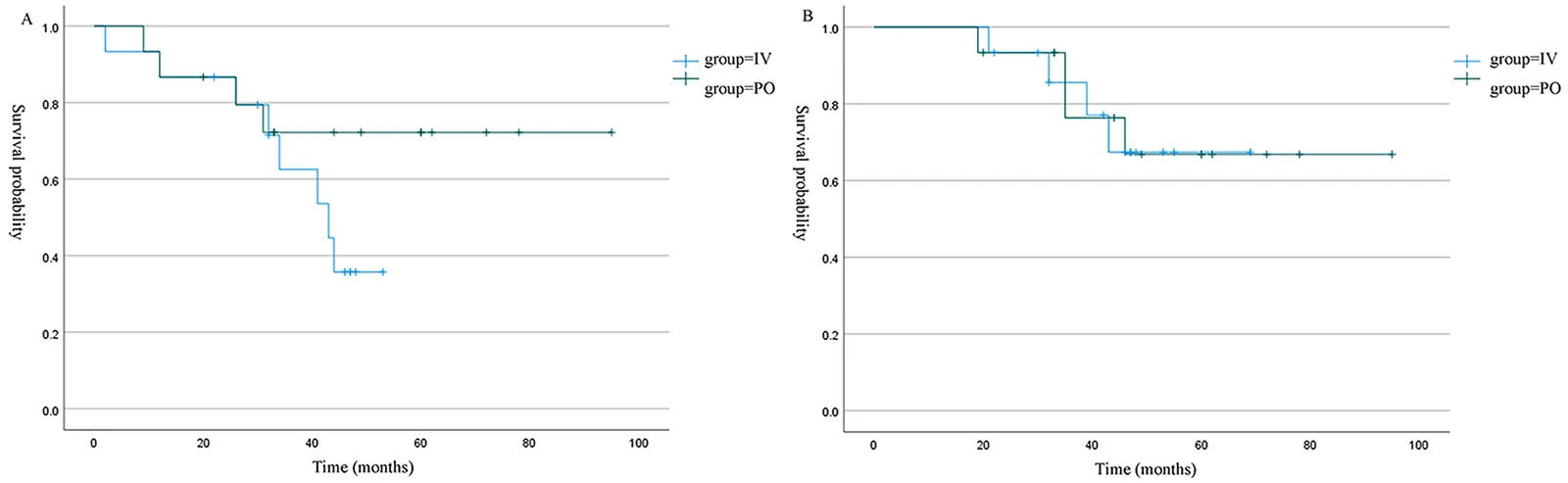
Kaplan-Meier estimates of progression-free survival **(A)** and overall survival **(B)** in multiple myeloma patients according to melphalan formulation (IV vs. PO).

## 4 Discussion

In recent years, immunomodulatory agents, monoclonal antibodies, and small-molecule targeted therapies have improved remission rates and significantly improved long-term survival among patients with hematological malignancies. Nevertheless, ASCT remains integral and the conditioning regimen is a key determinant of transplantation outcomes. BEAM is the most used conditioning regimen for both NHL and HL, and high-dose melphalan may be considered as the conditioning regimen for ASCT in patients with MM. However, in many resource-limited regions of Asia, including China, intermittent drug shortages and high treatment costs associated with IV melphalan persist as real challenges. Currently, there is a significant lack of real-world comparative data-adjusted for PSM-that evaluates PO versus IV melphalan in Asian populations, covering both lymphoma (BEAM regimen) and MM. This study provides matched, adjusted real-world evidence, focusing on hematopoietic recovery, differences in toxicity profiles, and survival outcomes. Although PO melphalan cannot replace IV formulations as the standard therapy, these findings offer valuable insights for clinical informed consent, supportive care planning, and future prospective pharmacokinetic trials in centers where access to IV melphalan is limited.

This study is one of the first comparisons of the long-term efficacy and safety of oral versus IV melphalan as components of conditioning regimens for ASCT in Chinese patients with lymphoma and MM. Although comparable short-term clinical outcomes were demonstrated, the findings do not establish pharmacokinetic equivalence or bioequivalence between formulations, which is a significant limitation given the well-documented and highly variable bioavailability of oral melphalan. The association between severe anemia and delayed PLT engraftment in the PO group was analyzed, and the divided-dose schedule of oral melphalan was associated with lower reported gastrointestinal toxicity. These findings support the rational use of oral melphalan as an alternative to IV melphalan.

### 4.1 Potential mechanisms underlying differences in hematopoietic reconstitution and toxicity

IV engraftment times were consistent with published data (16, 17). In contrast, delayed hematopoietic recovery was observed in the PO group, with significantly longer times to neutrophil engraftment in patients with lymphoma (12.00 vs 10.00 days, *p* = 0.025) and to PLT engraftment in patients with MM (14.00 vs 11.00 days, *p* = 0.021). These findings suggest that oral melphalan may cause prolonged drug exposure from extended gastrointestinal absorption, sustaining bone marrow suppression, which need basic research to confirm. In addition, reduced or incomplete drug delivery because of vomiting may also lead to delayed hematopoietic recovery. Pharmacokinetic variability (bioavailability, 20–60%) may further contribute to heterogeneous hematopoietic recovery. Higher grade 3–4 anemia in the PO group may reflect delayed bone marrow recovery and represents a notable difference from IV melphalan. The divided-dose schedule may reduce peak melphalan concentrations, contributing to lower incidence of gastrointestinal toxicity, including oral mucositis and diarrhea, observed in the PO group compared with the IV group.

Toxicity profiles differed between groups. Grade 3–4 anemia was more frequent in PO than IV (37.74% vs 18.87%; *p* = 0.031), whereas febrile neutropenia was less frequent in the PO group (35.85% vs 56.60%; *p* = 0.032). Prolonged exposure to lower drug concentrations may differentially affect hematopoietic cell lineages, with erythropoiesis more susceptible to sustained myelosuppression. The lower febrile-neutropenia rate despite slower neutrophil recovery might be associated with that the different ways of drug exposure result in different toxic spectra. In contrast, significantly higher rates of oral mucositis (33.96% vs 7.55%; *p* = 0.001) and diarrhea (37.74% vs 18.87%; *p* = 0.031) were observed in the IV group. Consistent with the findings of Vogl et al., a meeting reported an association between melphalan exposure and mucositis severity, higher IV peak concentrations may increase mucosal toxicity while potentially enhancing antitumor activity (18). This interpretation is supported by the observation that most patients who developed mucositis were in CR at the time of transplantation (83.33% in the IV group and 100% in the PO group; **Supplementary Table S1**), suggesting a possible association between mucosal toxicity and improved disease control. Given the small sample size of the mucositis subgroup (IV, *n* = 18; PO, *n* = 4), these findings should be interpreted with caution.

The divided oral dosing schedule (lymphoma: 140 mg/m² administered as two divided doses on day −1; MM: 200 mg/m² administered over 2 days, with each daily dose further divided) was adopted based on practical considerations and prior reports to reduce gastrointestinal toxicity (14, 19). However, oral melphalan bioavailability is highly variable and may decrease with repeated dosing, contributing to interpatient variability (14).

This study showed lower incidences of diarrhea (18.87% vs 37.74%) and oral mucositis (7.55% vs 33.96%) with the divided oral dosing schedule, yielding overall gastrointestinal toxicity rate (defined as the occurrence of at least one of these events) of 21.7% in the PO group compared with 56.60% in the IV group. This supports the divided-dosing rationale. However, the effect of repeated dosing on oral melphalan bioavailability requires pharmacokinetic studies and direct comparison with IV melphalan.

A key limitation of this study was the absence of pharmacokinetic assessments, which precluded comparison of drug exposure between formulations. The well-documented variability in the bioavailability of oral melphalan (20–60%) precludes its consideration as bioequivalent to IV melphalan. Comparable clinical outcomes may reflect differences in drug exposure rather than equivalent systemic exposure.

### 4.2 Interpretation of survival outcomes and comparison with existing literature

Despite slightly delayed PO engraftment, no significant differences were identified between the two routes of administration with respect to CR rates, PFS, or OS. These findings suggest that, within this follow-up period, melphalan formulation was not associated with compromised disease control or survival. In the lymphoma cohort, despite a significantly longer follow-up duration in the PO group than in the IV group (73 vs 42 months, *p* < 0.001), the estimated 3-year PFS rates (78.9% vs 81.6%, *p* = 0.773) and 3-year OS rates (89.5% vs 86.8%, *p* = 0.722) were comparable (**Table 7**).

In the MM cohort, where follow-up durations were balanced between the groups, the estimated 3-year PFS rates (66.7% vs 73.3%, *p* = 1.000) and 3-year OS rates (86.7% vs 80.0%, *p* = 0.672) were likewise comparable (**Table 8**).

These findings are consistent with Shen et al., who found no significant survival difference between oral and IV melphalan (6). However, they differ in part from Vela-Ojeda et al., who reported that IV melphalan as an independent predictor of DFS (RR = 1.4, *p* < 0.05) and OS (RR = 1.4, *p* < 0.01) in multivariable analysis, particularly among transplant recipients in objective response (CR/VGPR/PR) (8). This discrepancy may reflect differences in patient populations, sample sizes, follow-up duration, or the potential confounding effect of post-transplant maintenance therapy. It is worth noting that there have been real-world and clinical trial comparisons of the pre-treatment dose of 140 mg/m² versus 200 mg/m² of melphalan for multiple myeloma. The results showed that there was a very small difference in the time from progression-free survival to the next treatment for both doses, suggesting that fluctuations in the dose of melphalan within a certain range have a limited impact on the long-term disease control endpoint. This result provides a reasonable explanation for this study: even if there is a 20-60% individual bioavailability difference in oral melphalan, as long as the drug exposure in the body remains above the minimum effective treatment threshold, it is unlikely to cause a significant decline in clinical efficacy. However, this study lacks individual pharmacokinetic data, and this hypothesis still needs to be verified (20–22). In this study, all patients achieved at least PR before ASCT, and most achieved CR. In contrast, 57% of patients in the study by Vela-Ojeda et al. received maintenance therapy with interferon or thalidomide, which may have differentially influenced survival outcomes.

Multivariable Cox regression analysis (**Table 9**) showed that melphalan route was not an independent predictor of PFS or OS in either the NHL cohort (PFS: HR = 0.91, 95% CI 0.52–1.59, *p* = 0.735; OS: HR = 0.88, 95% CI 0.48–1.61, *p* = 0.681) or the MM cohort (PFS: HR = 0.79, 95% CI 0.43–1.45, *p* = 0.442; OS: HR = 0.81, 95% CI 0.42–1.56, *p* = 0.525). In contrast, established prognostic factors, including pre-ASCT CR status in NHL and high-risk cytogenetics in MM, remained significant predictors of outcome. These findings differ from those reported by Vela-Ojeda et al., in whom IV melphalan administration was identified as an independent predictor of both DFS (RR = 1.4, *p* < 0.05) and OS (RR = 1.4, *p* < 0.01) in patients with MM (8). This may reflect sample size differences (MM cohort in this study, *n* = 30; Vela-Ojeda et al., *n* = 52), follow-up, or the higher proportion of patients who were in CR at the time of transplantation in the present study (33–40% vs 25% in the study by Vela-Ojeda et al.), which may have attenuated the relative benefit associated with the IV formulation. These findings are consistent with prior studies identifying pre-transplant disease status as a key determinant of post-ASCT outcomes in patients with lymphoma (23). Age predicted both PFS and OS in the NHL cohort (**Table 9**), consistent with its established prognostic role of age in patients undergoing high-dose therapy and ASCT (24). However, given lymphoma subtype heterogeneity, further studies are warranted to assess the impact of melphalan formulation in specific populations, such as patients with primary CNS lymphoma, where distinct prognostic factors may influence clinical outcomes.

**Table 9:** Multivariable cox regression analysis.

Cohort	Variable	PFS		OS	
		HR (95% *CI*)	*p*–value	HR (95% CI)	*p*–value
NHL (n=56)	Administration Route (PO vs IV)	0.91 (0.52–1.59)	0.735	0.88 (0.48–1.61)	0.681
	Age (per 10–year increase)	1.21 (1.03–1.42)	**0.021**	1.18 (1.01–1.38)	**0.038**
	Sex (Male vs Female)	1.15 (0.78–1.69)	0.482	1.09 (0.71–1.67)	0.684
	Pre–ASCT CR Status (Yes vs No)	0.42 (0.23–0.77)	**0.005**	0.38 (0.19–0.76)	**0.006**
	Disease Type (T/NK vs B–cell)	1.38 (0.95–2.01)	0.092	1.42 (0.92–2.19)	0.112
MM (n=30)	Administration Route (PO vs IV)	0.79 (0.43–1.45)	0.442	0.81 (0.42–1.56)	0.525
	Age (per 10–year increase)	1.09 (0.88–1.35)	0.425	1.12 (0.89–1.41)	0.329
	Sex (Male vs Female)	1.21 (0.76–1.93)	0.421	1.18 (0.71–1.96)	0.521
	IgG Type (Yes vs No)	1.48 (0.95–2.31)	0.082	1.52 (0.92–2.51)	0.101
	High–risk Cytogenetics (Yes vs No)	1.92 (1.13–3.26)	**0.016**	2.05 (1.15–3.66)	**0.015**

Models were adjusted for all variables with SMD ≥ 0.2 and clinically recognized prognostic factors. Multivariable analysis was not performed for the HL cohort due to the limited number of events. HR, hazard ratio; CI, confidence interval. Bold *p*–values indicate statistical significance (*p* < 0.05).

### 4.3 Strengths and limitations of the study

This study has several strengths, including a systematic comparison of oral and IV melphalan in an Asian population, PSM to reduce baseline confounding, and outcomes in lymphoma and MM. These provide preliminary real-world evidence on oral melphalan in resource-constrained settings.

However, several limitations should be considered. First, high-dose melphalan conditioning evidence derives predominantly from IV studies. Oral formulation is a pragmatic adaptation to drug shortages and cost constraints, not an evidence-based alternative from prospective clinical trials.

Second, the retrospective, non-randomized design introduces selection bias, as assignment was influenced by patient preference, economic factors, and drug availability. PSM was applied, residual confounding cannot be excluded, as shown by post-matching imbalances observed in several variables (e.g., sex in NHL, SMD = 0.54; time to transplantation in HL, SMD = −0.38; M-component type in MM, SMD = −0.58). Unmeasured factors, such as socioeconomic status and nutritional status, may have differed systematically between the groups.

Third, pharmacokinetic data were absent, a major limitation. The well-documented variability in the bioavailability of oral melphalan precludes assessment of whether comparable outcomes were achieved despite differences in drug exposure and prevents determination of whether the observed differences in toxicity profiles (e.g., a higher incidence of anemia in the PO group and a higher incidence of mucositis in the IV group) were related to systemic exposure, peak drug concentrations, or exposure duration.

Fourth, statistical power was limited, particularly in the MM (*n* = 30) and HL (*n* = 20) subgroups, increasing the risk of type II error and reducing the reliability of subgroup analyses (e.g., the association between diarrhea and survival outcomes). At least 98 patients were required to detect a hazard ratio of 1.4; however, the MM subgroup (*n* = 30) and HL subgroup (*n* = 20) both had statistical power below 70%. Therefore, all subgroup analyses including the observed difference in PLT engraftment time among patients with MM, the association between anemia and delayed PLT engraftment in the PO group, and survival comparisons across subgroups should be considered exploratory and not statistically definitive. These findings should not inform clinical decisions and require confirmation in larger prospective studies.

Fifth, the imbalance in follow-up duration within the lymphoma cohort (73 vs 42 months, *p* < 0.001) may have affected long-term outcome comparability, although the estimated 3-year PFS and OS rates were similar between the groups.

Sixth, the single-center design may limit the generalizability to other populations or healthcare settings with different patient characteristics, transplantation practices, or supportive care protocols.

Seventh, Pharmacokinetic assessment of oral melphalan was not performed and oral melphalan should not be a bioequivalent substitute for the IV formulation and should be reserved for selected patients following informed consent regarding pharmacokinetic uncertainties and with close clinical monitoring.

Eighth, confounding in toxicity assessment, treatment strategies, and supportive care should be done according to disease and conditioning regimen in the future prospective study.

Finally, 1:1 PSM with a limited sample risks overmatching and reduced statistical power. Only clinically relevant covariates were included in the matching model, and residual imbalances (SMD ≥ 0.2) were adjusted for in multivariable analyses. Given the exploratory nature of the study and the reduced sample size after matching, the findings, particularly those derived from subgroup analyses, should be interpreted with caution.

In summary, although this study provides valuable real-world data on oral melphalan for ASCT conditioning, the limitations indicate that the findings should be hypothesis-generating rather than definitive. Validation in larger prospective multicenter studies with pharmacokinetic assessment is required to define the role of oral melphalan and identify optimal dosing strategies and patient selection criteria.

### 4.4 Implications for future research and clinical practice

Despite comparable clinical outcomes, oral melphalan cannot be a replacement for IV melphalan as the standard of care for several reasons. First, existing guidelines are based on clinical trials using IV melphalan, and the only multivariable analysis comparing the formulations identified IV melphalan as an independent predictor of survival (8, 25–27). Second, IV pharmacokinetics are predictable, whereas bioavailability of oral melphalan is highly variable (20–60%) (14, 15). Third, pharmacokinetic data are absent, precluding confirmation of therapeutic equivalence. Fourth, the oral formulation has not been prospectively validated in this clinical setting.

However, where IV melphalan is unavailable or unaffordable, and oral melphalan may reduce treatment costs by approximately RMB 40,000–50,000, oral melphalan may be a pragmatic alternative enabling access to ASCT for selected patients. The following measures should be considered: (1) careful patient selection and comprehensive informed consent regarding the off-label use of oral melphalan and the uncertainties associated with its pharmacokinetics; (2) therapeutic drug monitoring, where feasible, to facilitate individualized dosing, although validated therapeutic targets for oral melphalan are not yet established; (3) close monitoring of hematopoietic recovery accompanied by appropriate supportive care; and (4) systematic data collection to strengthen the evidence base.

Future research should prioritize prospective multicenter studies with pharmacokinetic assessments (e.g., AUC) to establish efficacy when compared to IV melphalan, define optimal dosing strategies, identify patients most likely to benefit, and assess therapeutic drug monitoring-guided dosing approaches. Until such data are available, oral melphalan should be used with caution and reserved for selected patients in whom ASCT benefits outweigh the uncertainties of the oral formulation.

## 5 Conclusion

This single-center retrospective cohort study showed that, when IV melphalan is unavailable or unaffordable, oral melphalan used as a conditioning regimen for ASCT was associated with slightly delayed PLT engraftment in patients with MM and a higher incidence of grade 3–4 anemia, while yielding comparable 3-year survival outcomes in patients with lymphoma and MM undergoing ASCT. The divided-dose schedule of oral melphalan was associated with a lower incidence of gastrointestinal toxicity, and the higher incidence of grade 3–4 anemia observed in the PO group was significantly associated with delayed PLT engraftment. Multivariable analysis further demonstrated that the route of melphalan administration was not an independent predictor of survival in this cohort. However, given variable bioavailability of oral melphalan (20–60%), the absence of pharmacokinetic data, the retrospective study design, and the limited sample size of certain subgroups (MM subgroup, *n* = 30, with insufficient statistical power), these findings should be interpreted with caution. Oral melphalan should not be a bioequivalent substitute for the IV formulation and should be reserved for selected patients following informed consent regarding pharmacokinetic uncertainties and with close clinical monitoring. Prospective multicenter studies incorporating pharmacokinetic assessment are needed to validate these findings.

## Data Availability

The original contributions presented in the study are included in the article/**Supplementary Material**. Further inquiries can be directed to the corresponding authors.
