# Supplementary material for: Oral versus intravenous melphalan for autologous stem cell transplantation conditioning in lymphoma and multiple myeloma: a retrospective cohort study
Source: Front Oncol. 2026 Oct 19;16:1923679. doi: 10.3389/fonc.2026.1923679 (PMC13724488; doi:10.3389/fonc.2026.1923679)
Supplement: Supplementary file 1 [file Table1.docx]

Table S1 Disease status of patients with oral mucositis

| disease status | IV group (*n* = 18) | PO group (*n* = 4) | *p*–value |
| --- | --- | --- | --- |
| CR, *n* (%) | 15 (83.33) | 4 (100.00) | 0.547 |
| PR, *n* (%) | 3 (16.67) | 0 (0.00) |  |

Table S2 Survival benefits in lymphoma patients of PO group with diarrhea

| Disease status after ASCT | Diarrhea (*n* = 10) | No diarrhea (*n* = 28) | *p*–value |
| --- | --- | --- | --- |
| CR, *n* (%) | 9 (90.00) | 22 (78.58) | 0.565 |
| PR, *n* (%) | 1 (10.00) | 3 (10.71) |  |
| Progression, *n* (%) | 0 (0.00) | 3 (10.71) |  |

Table S3 Survival benefits in MM patients of PO group with diarrhea

| Disease status after ASCT | Diarrhea (*n* = 1) | No diarrhea (*n* = 14) | *p*–value |
| --- | --- | --- | --- |
| CR, *n* (%) | 0 (0.00) | 6 (42.86) | 0.626 |
| PR, *n* (%) | 0 (0.00) | 1 (7.14) |  |
| VGPR, *n* (%) | 1 (100.00) | 7 (50.00) |  |
| Progression, *n* (%) | 0 (0.00) | 0 (0.00) |  |
